# Single step synthesis of Schottky-like hybrid graphene - titania interfaces for efficient photocatalysis

**DOI:** 10.1038/s41598-018-26447-9

**Published:** 2018-05-25

**Authors:** Zhifeng Yi, Andrea Merenda, Lingxue Kong, Aleksandra Radenovic, Mainak Majumder, Ludovic F. Dumée

**Affiliations:** 1 Deakin University, Geelong, Institute for Frontier Materials, Waurn Ponds, 3216 Victoria, Australia; 2 Ecole Polytechnique Federale de Lausanne (EPFL), Institute of Biotechnology, CH-1015 Lausanne, Switzerland; 3 Department of Mechanical and Aerospace Engineering, Nanoscale Science and Engineering Laboratory (NSEL), Monash University, Clayton, 3800 Victoria, Australia; 4 ARC Research Hub for Graphene Enabled Industry Transformation, Monash University, Clayton, Victoria, Australia

## Abstract

The development of 2D nanomaterial coatings across metal surfaces is a challenge due to the mismatch between the metal microstructure and the nanoscale materials. The naturally occurring thin oxidative layer present across all metal surfaces, may lead to low adherence and connectivity. In this paper, graphene/titania/Titanium hybrid films were for the first time fabricated by a single step chemical vapour deposition process across Titanium foils. The presence of graphene as a dopant was found to enhance the photocatalytic performance of the final products, applied to the degradation of organic molecules and to lead to Schottky-like junction formation at the metal/oxide interface. These Schottky junctions, where vacancies are present across the titania material due to the graphene doping and where Ti^3+^ ions are predominantly located, yield enhanced catalytic performance. The highest degradation rate was found to be 9.66 × 10^−6^ min^−1^, achieved by the sample grown at 700 °C for 5 min, which was 62% higher than the sample just treated at that temperature without graphene growth. This work provides evidence that graphene may be grown across pure Titanium metal and opens new avenues in biomedical devices design, tribological or separation applications.

## Introduction

Graphene materials, composed of stacked one-atom thick and two dimensional (2D) sp^2^-bonded hexagonal carbons, have attracted attention due to their outstanding native phonon conduction properties^1–3^. The potential of graphene nano-composite materials with enhanced surface properties has been widely demonstrated in the areas of super-capacitors, energy storage devices, sensors, membranes, anti-corrosion coatings and electrode-materials to name a few^1–11^.

Graphene fabrication processes include (i) epitaxial growth, (ii) mechanical cleavage of graphite, (iii) reduction of graphene oxide (GO), and (iv) chemical vapour deposition (CVD)^12–15^. Although the CVD method is one of the most versatile techniques to synthesize graphene grains with controllable surface area and number of layers^16^, challenges to control the substrate/graphene interface and interactions still remain^16^. The growth mechanism and the impact of the different growth parameters, including the carbon source, the temperature and growth duration on the graphene quality, are highly related to synergistic interactions with the microstructure and roughness of the growth surface^17^.

Substrates for graphene growth by CVD primarily include metal materials, such as copper, nickel, aluminium and stainless steel^17,18^. These metals can perform both as catalysts and as substrates for graphene growth, therefore leading to either direct deposition of graphene or doping of the metal/metal oxide surface grains^19^. A major challenge in the CVD growth of graphene coatings is the ageing of the metal exposed for extensive periods at high temperatures. This effect may alter the microstructure and grain distribution across the metal as well as affect the bulk electronic or mechanical properties of the carbon-metal interface^18^.

Although Titanium (Ti) and related alloys, as a promising material for their corrosion resistance, biocompatibility, light-weight and photocatalytic properties, have been extensively studied^20^, novel growth strategies to deposit and atomically bond graphene to Ti are lacking. A recent study demonstrated that graphene can be directly coated on a Ti based alloy (Nitinol (NiTi 50/50)) through CVD, and the graphene layers were found to enhance the bioactivity of the metal surface in terms of cytoskeleton development and of spontaneous osteogenic differentiation of Mesenchymal stem cells^21^. Although Nitinol is a material more temperature – stable than pure Ti, the potential release of nickel metal from nitinol alloys is however always an issue for medical application^22^, compared to pure Ti products. Direct surface functionalization or decoration across pure Ti is also seldom reported, which may be primarily attributed to the low catalytic property and high chemical stability of the Ti metal, which strongly restricts surface functionalisation^21^. Therefore, sol gel pathways and the implementation of versatile 2D nanoscale coatings, such as graphene, across the surface of a metal as an active and functional layer may bring new functionalities and applications to graphene hybrid semiconductors and related Ti/graphene materials in the photocatalysis and photosynthesis areas^23–27^.

Herein, a one-step graphene CVD growth process across pure Ti substrates is demonstrated for the first time to generate hybrid graphene/TiO_2_/Ti materials. The growth of graphene across the Ti template was favoured though the controlled injection of a hydrogen/acetylene mixture, limiting the titania formation over the seeding steps of the growth at low temperature. The impact of temperature, growth duration and surface roughness of the Ti templates were systematically correlated to the quality and homogeneity of the graphene coatings. Furthermore, photocatalytic performance of the combinatorial hybrid materials was investigated through the degradation of a model organic dye molecule. The presence of the graphene doping across the surface of the materials was found to enhance the bare TiO_2_ photocatalytic properties by simultaneously facilitating electrons mobility from TiO_2_ to graphene as well as doping the anatase lattice of the surface titania shifting the band gap of the materials, providing Schottky-like behaviours.

## Experimental

### Materials

Commercial roll formed Ti foils (purity > 99.6%) with a thickness of 0.1 µm were purchased from Goodfellow Cambridge Ltd. Rhodamine 6 G (R6G) were purchased from Sigma Aldrich. Hexane (99%) were purchased from Sigma Aldrich and its vapor was used as carbon source for graphene growth. The other gases used for the CVD growth were a mixture of argon (Ar)/hydrogen (H_2_) (85/15 at.). All chemicals were analytical grade and used without any further purification.

### CVD growth of graphene

The growth of graphene across the Ti foils was performed in a low pressure CVD set-up with a tube furnace from Lenton (LTF 14/50/450), following a previously demonstrated procedure by our group^18^. Before graphene growth, Ti foils were first cut into a size of 2 cm in length and 1 cm in width prior to being loaded in a quartz boat within the tube furnace. The whole system was pumped down to a target pressure below of 200 mTorr for 30 min to remove any adsorbed remaining oxygen gas molecules. Subsequently, a mixture of Ar/H_2_ (85/15) was used to purge and flush the tube and samples. The pressure was then set with these gases at 4 Torr for 15 min with a flow rate of 400 cubic centimetre per minute (ccm). Under the Ar/H_2_ gas, a series of samples were treated by adjusting the growth temperature (700, 900 and 1000 °C) and duration (2, 5 and 10 min). The hexane flow rate was maintained at 20 ccm for all tests since previously found to lead to the highest quality of graphene in that similar CVD system^18^. During the growth duration, the pressure in the tube was maintained at 4 Torr and after growth, the samples were naturally cooled under vacuum which is a quenching process to facilitate the growth of TiO_2_. After the temperature decreased to less than 100 °C, samples were removed from the tube and stored at room temperature for further use.

The surface of Ti foils was polished using the following standard mechanical polishing procedure to investigate the impact of surface roughness on the growth of graphene. The first step involved grinding the sample on 1200 grit SiC paper for 1 min, followed by 3 and 1 micron diamond powder polishing for 5 and 7 min, respectively. The growth conditions across the polished samples were kept the same as these were performed across the plain, unpolished, Ti foils.

### Characterisation techniques

Scanning electron micrographs (SEM) were captured on a JSM 7800 F FEG-Scanning Electron Microscope (JEOL, Japan). The tests were performed on un-coated samples as provided with a 5 keV accelerating voltage at a distance of 10 mm without prior coating. Raman spectra were obtained on an inVia Raman microscope (Renishaw, United Kingdom) at the laser wavelength of 514 nm. An extended scan ranging from 100 to 4000 cm^−1^ was performed for 10 s with laser power of 25 mW. X-ray photoelectron spectroscopy (XPS) were performed on a K-Alpha X-ray photoelectron spectrometer (Thermo Fisher Scientific, Australia). A quantitative elemental composition of Ti, oxygen and carbon for a surface depth of 1–10 nm was carried out. The technique could detect elements with a detection limit of 0.1% of the bulk material. An Al Kα (1486.6 eV) X-ray source was used as the excitation source, and the anode was maintained at 250 W, 10 kV and 27 mA at a chamber pressure of (1 ± 0.1) × 10^−9^ Pa with an oval beam spot size of 400 μm × 400 μm. In a typical setup, the X-ray source was set at 45 μm from the sample surface while the angle between the analyser and the sample surface is 90°. The analysis was performed on 3 different spots on each sample, under the same experimental conditions. The spectra were acquired from 10 cumulative scans, for a pass energy of 152.1 eV and a dwelling time of 0.5 s. The high-resolution spectra for C 1 s, O 1 s and Ti 2p were acquired at a pass energy of 20 eV and 20 cumulative scans. The spectra were then deconvoluted using the CasaXPS software and the high resolution peaks fitted with a mixed Gaussian-Lorentzian function (GL30), as previously reported^28^, while the asymmetric nature of the sp^2^ C in the C 1 s spectra was fitted with a Gaussian-Lorentzian product formula modified by an asymmetric form A(0.1, 0.1, 80)GL(80) to account for its asymmetric tail^29^. The asymmetric shape of Ti (0) in the Ti 2p spectra is fitted with a LA(1.1,5,7)^30^. X-ray diffraction (XRD) patterns were collected on a X’Pert Powder instrument (PANalytical, Netherlands) using a Cu Kα radiation source (λ = 1.54181 Å) operated at 40 kV with a 30 mA current. XRD data were recorded over a range of 6–70°. The ultraviolet visible (UV-Visible) spectra were obtained from USB-2000 ultraviolet visible spectrometer (Ocean Optics, United States). The R6G absorbance peak at the wavelength of 526 nm under UV-Vis was used to determine the concentration over time. The UV-Visible Diffuse Reflectance Spectroscopy (DRS) was conducted on a LAMBDA 1050 UV-Vis spectrophotometer (Perkin Elmer, United States) equipped with an integrating sphere. Prior to the analysis, the reflectance port was removed to take off the contribution of the specular reflection from the total reflectance, only the diffuse reflectance was consequently collected. The reflection was calibrated on a Spectralon standard, and the calculated %R is referred to this standard. The data was acquired with a UV WinLab software at a fixed scan speed of 485.33 nm/s, and the Kubelka Munk function subsequently determined.

### Photocatalytic performance

As a model molecule, R6G was selected to test the photocatalytic performance of Ti foils with or without graphene growth. A stock solution of R6G was prepared at a concentration of 7.5 mg/L. For each test, 2 mL of R6G solution was transferred to a quartz cuvette and the sample with an area of around 25 mm^2^ was placed on the bottom of the cuvette, which was exposed to UV light generated from an OmniCure S2000 UV lamp (Excelitas Technologies, United States) to induce the degradation of R6G. The intensity of UV light is 250 mJ/cm^2^ with the wavelength, set with a grated plate filter, between 320 and 480 nm and the sample to lamp distance is 10 cm. The degradation of R6G was determined through measuring the peak absorbance of R6G at the wavelength of 500 nm at different time intervals.

## Results and Discussion

Graphene/TiO_2_/Ti hybrid materials were investigated in terms of their morphology, structure, and photocatalytic properties, to not only understand the growth mechanism, but also evaluate synergistic properties and the role of graphene doping on the band gap of the semiconductor. A schematic of the growth process is shown in Figure SX.

**Figure 1:**
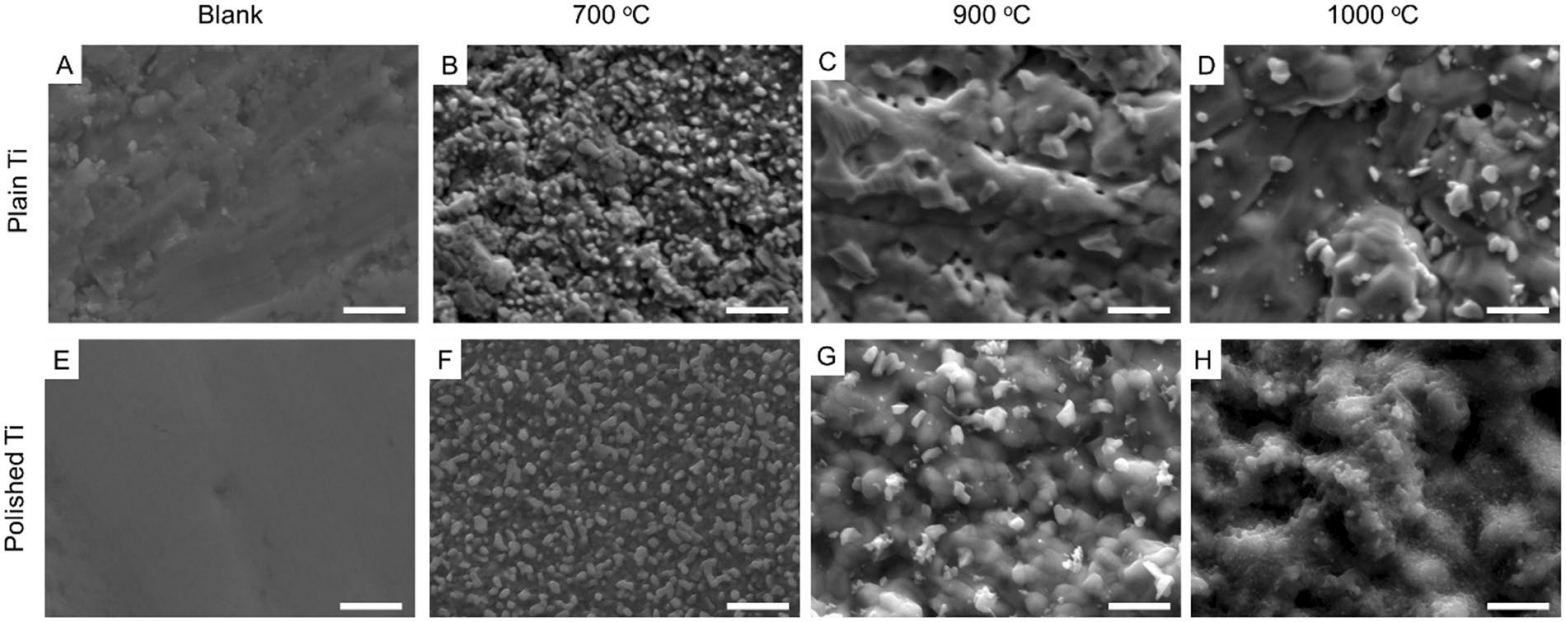
SEM images of Ti foils with different treatment. (**A**) Plain surface of Ti foils; (**B**–**D**) Plain Ti foils with graphene grown at different temperature. (**E**) Polished surface of Ti foils; (**F**–**H**) Polished Ti foils with graphene grown at different temperatures. Scale bars are 1 μm.

The morphology of the Ti foils at different temperatures and growth durations is shown in Fig. 1, Figures S2, S3 and S4, respectively. The polishing process greatly smoothened the surface of Ti foils as seen in Fig. 1A and E. A smoother structure can be seen on the polished surface with graphene grown at 700 °C (Fig. 1I) than the plain ones (Fig. 1C), but there were no significant differences across the series of other samples with graphene grown at higher temperature and for longer durations. At higher temperature, the Ti metal may start to soften leading to a smoothening of the surface, as seen across the SEMs of the samples treated at 900 and 1000 °C^31^. The hexane gas flow during graphene growth at the experimental temperature was also found to affect the surface morphology. Without any gas flow, there was noticeable large crystal-like structure formed in the heat-treated samples (Figures S2, S3 and S4) compared to the smoother surface with hexane flow, namely with graphene growth (Fig. 1B–D and F–H). These crystals were amorphous titania, which were likely generated upon H_2_ absorption by the Ti matrix during the heating phase of the CVD growth. Ti is known to act as a hydrogen sponge at temperatures around 650 to 700 °C making high temperature heat treatments challenging due to physical changes of the metal matrix and microstructure^32^.

**Figure 2:**
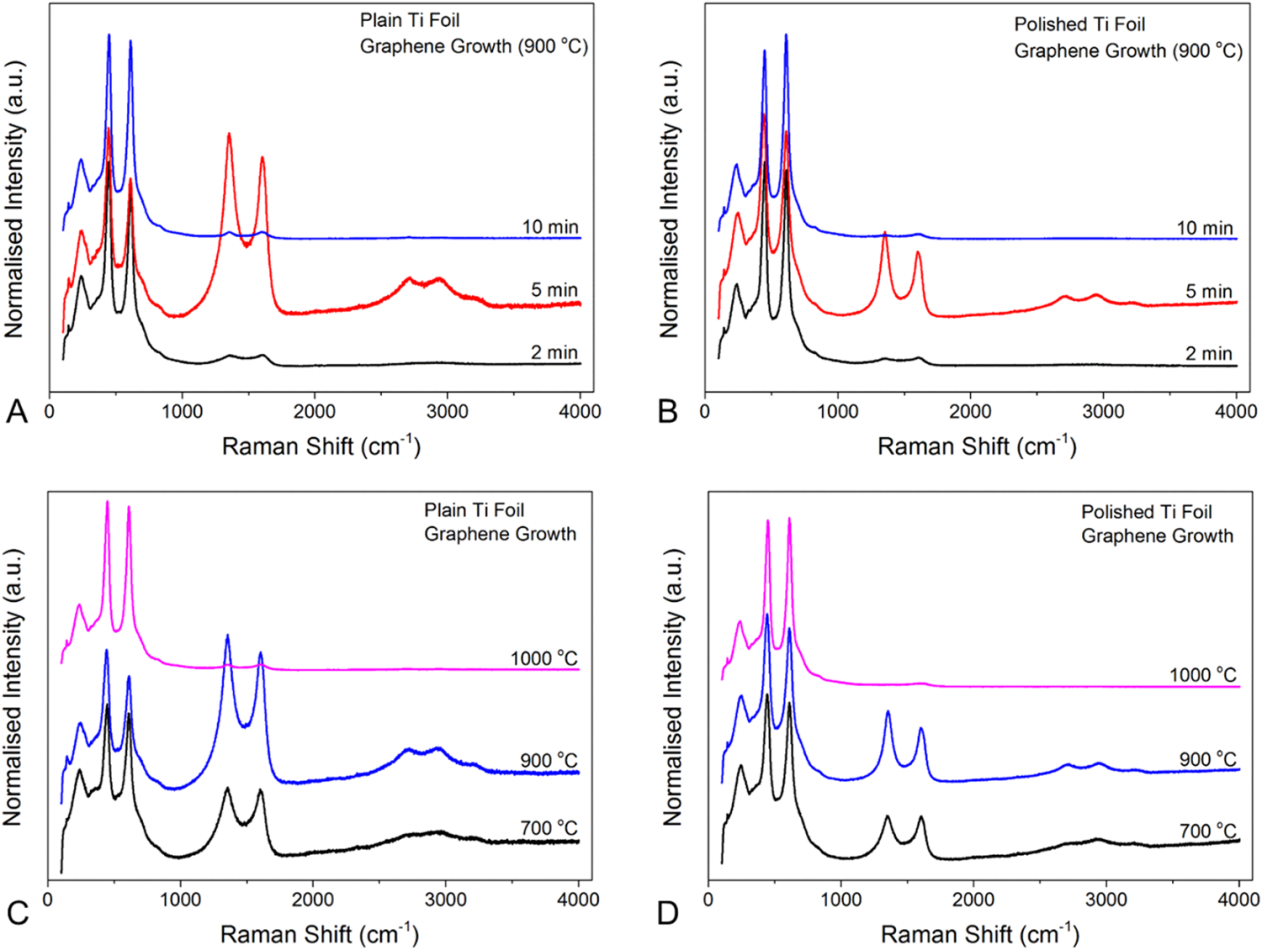
Raman spectra of Ti foils with graphene grown at different experimental conditions. Plain (**A**) and Polished (**B**) Ti foils with graphene grown at 900 °C for 2, 5 and 10 min. Plain (**C**) and polished (**D**) with graphene grown at different temperatures, 700–1000 °C, for 5 min.

The graphene component of hybrid graphene/TiO_2_ layer grown across the Ti foils was further investigated through Raman spectroscopy (Fig. 2 and Figure S5). The blank Ti foils with and without polishing were firstly examined through Raman where no obvious peak could be detected (Figure S6). For the samples with graphene growth, the D-band at around 1350 cm^−1^ and the G-band at around 1600 cm^−1^ are the representative peaks for graphene representing disorder-induced band of graphene and the graphitic band, respectively^33,34^. Also, the peak at around 2500–3200 cm^−1^ indicates that the 2D-band (secondary D-band) of graphene, which is related to the number of graphene layers^35,36^. The Raman results from the time series grown at 900 °C indicate that graphene can be clearly detected from 5 min of growth duration. For shorter growth durations, it is likely that the carbon atoms surface segregation leads a predominant role in the graphene growth mechanisms. As previously shown for catalytic surfaces similar to Ti/TiO_2_, such as Ni surfaces, carbon atoms shall first diffuse from the surface into the first few nanometers of the bulk of the growth support during the annealing stage and predominantly at high temperature. After the diffusion step, the carbon atoms shall precipitate and recombine onto the activated Ti catalyst during the cooling period^37^. Further improvements in the growth conditions to limit H_2_ uptake may include a sharper control of the moment when H_2_ gas is introduced, particularly upon reaching a set reactor temperature higher than 700 °C.

In the temperature series, the growth duration was fixed at 5 min and clear D-band and G-band can be seen across the spectra at 700 and 900 °C (Fig. 2C). At 1000 °C, on the other hand, these two peaks were not present suggesting a poorer graphitization potentially due to the higher activation energy of Ti surface at that temperature^38,39^. The 2D-band is clearly shown in the sample grown at 900 °C and the broad 2D-band indicates a thick graphene layer^21^. Similar trends and graphene quality were obtained for the polished Ti foils (Fig. 2D) suggesting that the macro-roughness of the material did not extensively affect the graphene growth mechanism. This aspect is important as it highlights the potential scalability for production of graphene dopant layers across Ti. Indeed, for most other metals, such as Cu or Ni, the surface state was found to play a significant role in the graphitization process. Growth on such metals therefore required extensive surface polishing to achieve good precipitation/recombination of carbon atoms and was found to greatly affect the interfacial properties, such electrical or thermal conductions^40^.

The impact of the heat treatment on the Ti microstructure was also investigated at the same temperatures (Figure S5). No graphene materials were formed in these control conditions, highlighting the need for the carbon precursor (Raman data in Figure S4). Amorphous carbons were however likely formed as shown by the broad D-band and G-band without 2D-band (Figure S5), which could result from contamination of residual carbons left in the furnace. The polished samples showed similar trends, and the polishing process does not lead to any significant structure changes of the deposited graphene. Regarding the TiO_2_ component, the peaks at 230, 440 and 610 cm^−1^ from Raman spectra (Figs 2 and S4) are representatives of the rutile structure, *E*_g_ and *A*_1g_, conforming to the tetragonal space group of *P42/mnm*^41^. For all the samples with or without graphene growth, there were no significant differences in terms of the peak positions and intensities upon altering the growth temperature and duration. The ratio between D-band and G-band (I_D_/I_G_) intensities can be used to assess the presence of defects across the graphene^34,42^. As can be seen from Table 1, the lowest I_D_/I_G_ ratio was achieved for the sample grown at 700 °C for 5 min, correlated with the overall carbon content, which was also the highest compared for this sample across the series, as indicated by the XPS results (Figure S7). The quality of graphene grown on Ti in this paper was not as good as the previous reports on copper and nickel^17,43^, which might be due to its higher melting point and low catalytic properties to decompose carbon source.

**Table 1:** D and G peak position and D/G ratio of graphene grown at different temperatures with or without surface polishing.

Samples	Temperature (°C)	Peak Positions		I_D_/I_G_
		D-band	G-band	
Plain Ti Foils	700	1349.03	1608.08	1.01
	900	1354.00	1600.88	1.12
	1000	1352.76	1608.08	1.04
Polished Ti Foils	700	1352.76	1608.68	1.00
	900	1354.00	1603.28	1.23
	1000	—	—	—

The chemical state and purity of the graphene layer was furtherly investigated by the evaluation of the high-resolution spectra of C 1 s. Although the main C sp^2^ peak situated at 284.4 eV (Tables S1 and S2) represents the main component, additional chemical states of carbon can be revealed and can be related to impurity due to the partial oxidation and loss of graphitic structure. Additional peaks can be associated to C-OH bonds (285.4 eV), C-O-C (286.4 eV), C=O (287.4 eV) and C-OOH (288 eV) functionalities, respectively^44–46^. Nonetheless, the main peak occurring at 284.4 eV accounts for more than 80% of C states and reflects the presence of graphitic bonds, in good accordance with the literature on graphene CVD growth^47,48^. Interestingly, the chemical states representing higher oxidation states as C=O and C-OOH functionalities increase with the treatment temperature, as shown in Figure S8, suggesting that extreme growth conditions can be detrimental to the purity of graphene. Additional carbonaceous species have been already identified in CVD-growth graphene studies^49^, and their presence attributed to oxygen intercalation^50^ upon air exposure. The evidence of higher O content at higher process temperature reported in Figures S7 and S8 can be therefore related to more extreme growth conditions that characterize the substrate and that can be detrimental to the purity of graphene, generating more defects that can further react upon air exposure. The presence of oxidized carbon species in the TiO_2_/Ti/graphene composite can be confirmed by the analysis of the O 1 s spectra in Figure S9. The main peak, located at 529.8 eV (Full Width at Half Maximum (FWHM) = 1.26) is representative of the Ti-O bond, nonetheless the presence of different O functionalities is clear from the occurrence of multiple peaks. Additionally, the presence of Ti-OH is also revealed by the peak 531.7 eV. This peak, however, can also be assigned, together with the peak at 533 eV, to C=O, C-OOH and COO functionalities^51^ as represented in Tables S3 and S4. The appearance of a stronger peak at 533 eV for the sample grown at 1000 °C is therefore in good accordance with its relative C 1 s spectra that showed the presence of more impurities in the graphene matrix. Furthermore, an additional shoulder in the O 1 s spectra occurs at 530.5 eV. The assignment of this peak is controversial, as it can be associated to O vacancies in a TiO_2_ matrix^52^ or to O in Ti-O-C bonds^53^.

In order to gain more insights into the potential generation of O vacancies, and consequently of Ti suboxides that are known to be beneficial in photocatalytic phenomena^54^, the Ti 2p spectra has been considered. The orbital split related to Ti(IV) 3/2p and 1/2p can be clearly shown at 458.6 eV (FWHM = 1.22 eV) and 464.4 eV (FWHM = 2.03 eV), respectively, in good accordance with the literature^30^. Two more oxidation states are however present, as Ti^3+^ and Ti(0), classified in Table S5. A higher concentration of Ti^3+^ species can be observed in Figure S10, representing Ti-700, due to the higher integrated area associated to fixed peak position and FWHM, 457.9 eV and 2.2 eV, respectively, for Ti^3+^ 2p 3/2, as shown in Table S6. The presence of a higher content of Ti suboxides is particularly relevant for applications in catalysis as it can facilitate the charge transfer and hinder the recombination of holes and electrons^55^, although these species are highly unstable and their generation and control is particularly challenging. The presence of these species supports the design of the Schottky-like diode by lower energy barrier to activation. This behaviour was attributed to the presence of a multi-oxide interfacial layer in the top layer, available to UV excitation, of the hybrid material, as previously demonstrated for Ti ant TiOx alloys^56^.

**Figure 3:**
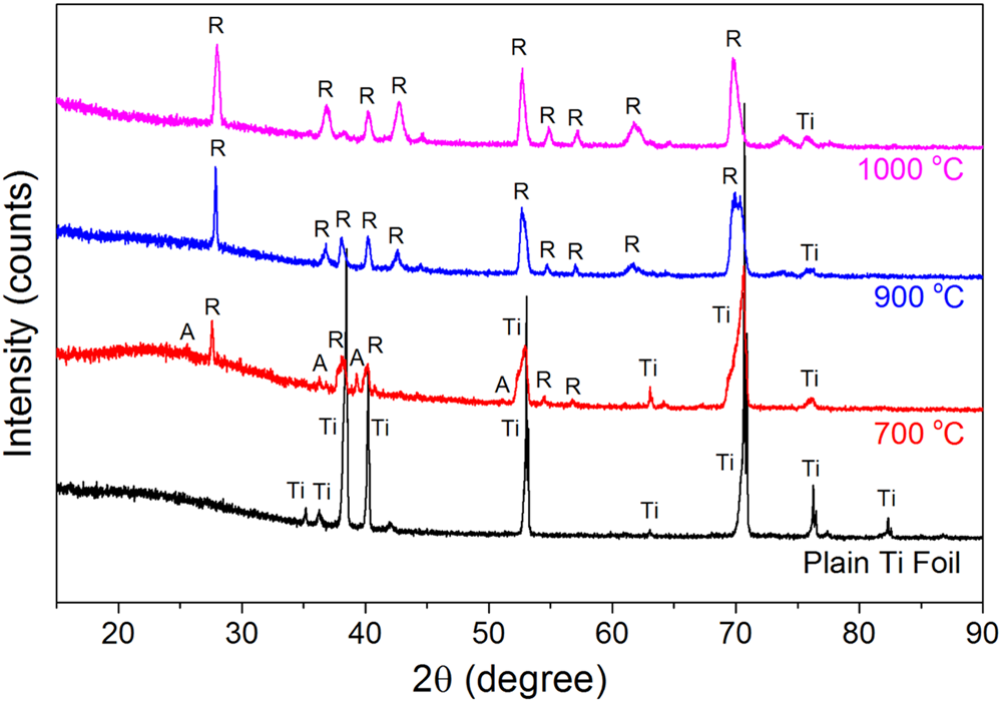
XRD profiles of the samples with graphene growth at different temperatures. Ti, A, R represent the characteristic peaks for Ti, anatase and rutile, respectively.

The crystal structure of TiO_2_ layer was further investigated by XRD (Fig. 3). The titania phase found for the samples grown at 900 and 1000 °C was fully assigned to a rutile structure. Interestingly, partial anatase structure contents were found for the samples grown at 700 °C along with rutile as the major component. This result is consistent with literature since the full transition temperature from anatase to rutile is typically reported at around 800 °C^31,57^. As anatase provides much higher photocatalytic properties than rutile, the photocatalytic performance of the samples was evaluated.

**Figure 4:**
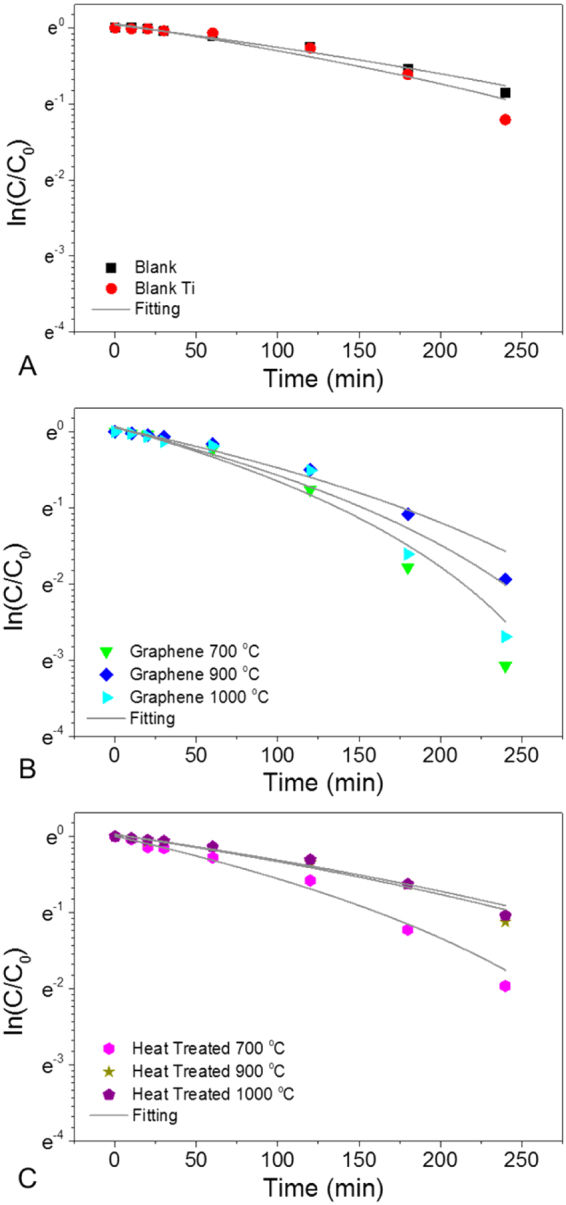
Photodegradation kinetics of R6G with graphene/TiO_2_/Ti hybrid materials over time. (**A**) Control group; (**B**) samples with graphene growth and (**C**) samples with only heat treatment. The continuous lines in all the graphs are the fitted curve using second-order reaction equation.

The photocatalytic performance of the graphene/TiO_2_/Ti hybrid materials was investigated by using R6G as a model molecule under UV light (wavelength up to 500 nm). The potential of graphene nanomaterials and as dopant was recently investigated in the form of quantum dots^58^ but also low dimensional semi-conductors^59,60^. The absorption spectra show that the absorbance peaks of R6G for all samples decreased with respect to exposure duration (Figure S11). The kinetics of catalysis is usually related to the diffusion of the dye molecules from the bulk solution to the surface of the catalyst, and thus a second-order reaction model was introduced to understand the degradation kinetics, as shown in Fig. 4. The kinetic constant was calculated through the following equation^61^:

1 $$\mathrm{ln}(\frac{{C}}{{{C}}_{0}})={k}{({a}-{t})}^{2}$$

where, *C*_0_ and *C* are the initial concentration of R6G and the concentration of R6G over time, *k* is the kinetic constant, *a* is a constant, and *t* is the reaction time. The fitting results for the UV-visible spectra are shown in Table 2. As suggested by the anatase content demonstrated in Fig. 3, the highest degradation rate was achieved for the sample grown at 700 °C for a growth duration of 5 min. The performance could also be attributed to the increased surface area achieved from the rough structure generated in these conditions as shown across the SEM images (Fig. 1C), offering larger contact area with the R6G molecules. Interestingly, the photocatalytic properties of the graphene/TiO_2_/Ti hybrid materials are 62%, 110% and 205% higher than those achieved by the control heat-treated samples at 700 °C, 900 °C and 1000 °C, respectively. This trend therefore indicate that graphene has a positive influence on the photodegradation properties of the Shottky-like surfaces.

**Table 2:** Calculated photodegradation constant and coefficients through second-order reaction equation.

Samples	k (×10^−6^ min^−1^)	R^2^
Blank (R6G solution)	1.96	0.97
Blank Ti foils	2.82	0.94
Graphene 700 °C	9.66	0.98
Graphene 900 °C	5.66	0.97
Graphene 1000 °C	7.58	0.96
Heat treated 700 °C	5.96	0.99
Heat treated 900 °C	2.69	0.98
Heat treated 1000 °C	2.48	0.98

Due to the high electron mobility of 2-dimensional graphene sheets^16^, combining graphene with TiO_2_ offers synergistic effects whereby electrons generated form the dye molecules may flow more freely from the TiO_2_ anatase to the graphene domains, thus generating Schottky-like barriers at the interface between TiO_2_ and conductive layers such as metal or graphene and due to the presence of the vacancies in the form of Ti^3+^ ions, leading to an enhanced charge separation. A Schottky barrier interface electric field between the Ti-metal and the Ti-oxides shall confine vacancies to the surface of the semiconductor as also observed across metal doped TiO_2_ catalysts^62^. Such interfaces may generate photoexcited electron-hole pairs and an electrostatic depletion layer at the junction between the metal-like graphene and the titania semiconductor, which causes the hybrid material to act as an electrical rectifier^63^. This process may thereby delay the charge recombination upon catalytic reactions^64,65^. The incorporation of graphene therefore offers outstanding doping capabilities for TiO_2_ photocatalytic properties enhancement. In addition, Ti left in the hybrid materials can also perform as a conductor to conduct electrons generated by TiO_2_ during the photocatalytic process.

The photocatalytic efficiency reported in this study is not as good as that of slurry system due to the lower exposed surface area that is available for photocatalytic reactions and low porosity of the supported catalyst layer^66–68^. However, these results are comparable with similar TiO_2_ based film system with the complete dye decomposition time between 3 and 4 h^69,70^. The presence of graphene doping the TiO_2_ matrix as well as vacancies as shown by the Ti^3+^ content in this hybrid materials facilitated the photocatalysis, despite of low anatase TiO_2_ content that is regarded to be more efficient than pure rutile while being applicable onto large surface areas of materials in a facile manner.

**Figure 5:**
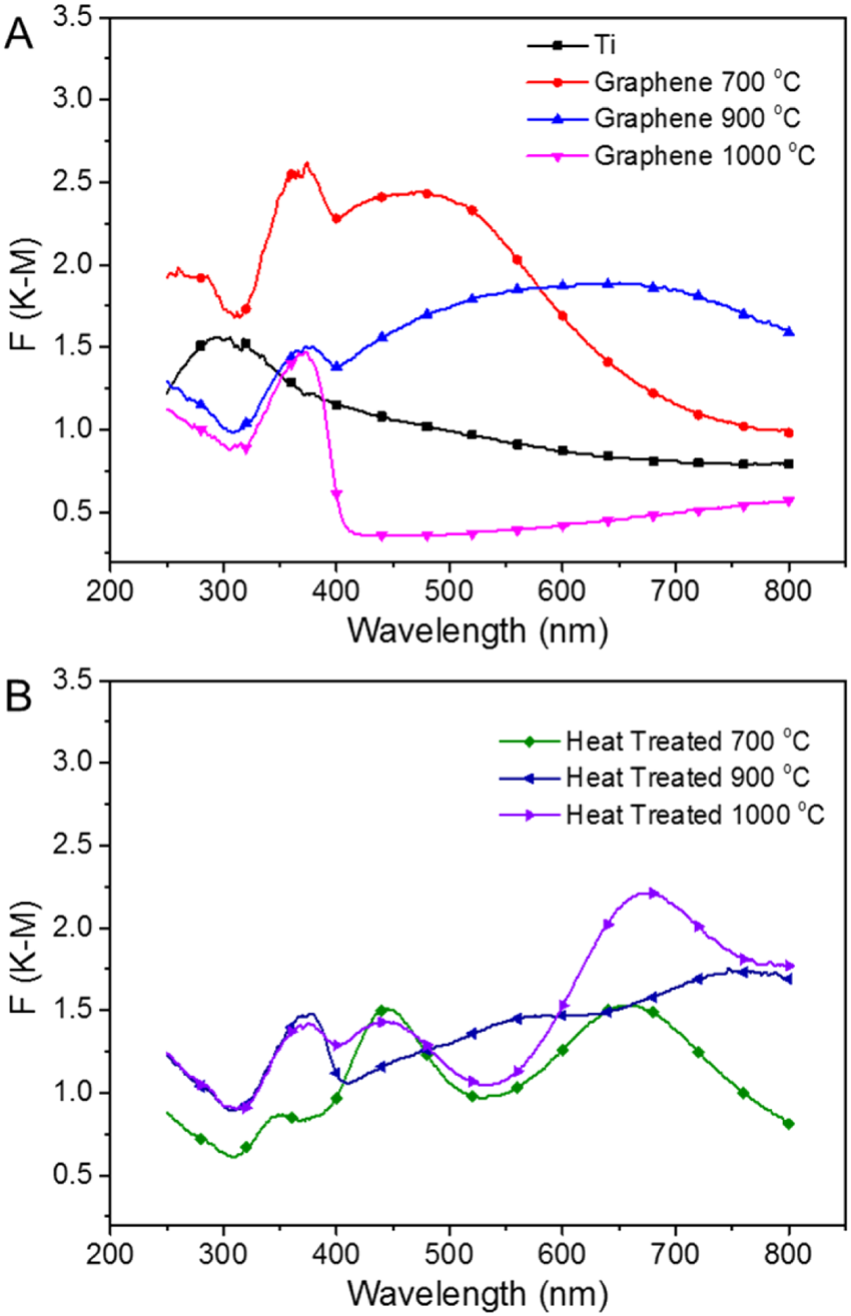
K-M plot of DRS for samples with different treatment. (**A**) plain Ti foil and samples with graphene growth at 700 °C, 900 °C and 1000 °C; (**B**) samples with heat treated at 700 °C, 900 °C and 1000 °C.

The impact of the doping was therefore further evaluated by DRS to evaluate the change in the band gap of the material. Figure 5 shows the results from DRS measurement related to the UV-Vis absorbance. As for the graphene/TiO_2_/Ti hybrid samples, the spectra in Fig. 5A shows a clear trend with a progressive increase of the absorption edge as the treatment temperature rises from 700 °C to 1000 °C. The sample treated at 700 °C presents an unconventional behaviour with two absorption peaks around 370 nm and 460 nm, respectively. These features may be referred to trapped holes and electrons on the TiO_2_, respectively, during the transition from anatase to rutile shown on the XRD results, which is in good agreement with other studies^71,72^. One would assume that plain TiO_2_ would absorb only in the UV-region as it has no activity under visible light due to the high band-gap, 3.2 for anatase and 3 for rutile, respectively. The presence of clear absorption features in the visible range is therefore inconsistent with the literature^73,74^. However, considering that the photocatalytic tests were conducted under a UV light with wavelength up to 500 nm, the outcomes of the analysis are in good accordance with these spectra demonstrating that the plain foils absorb radiation in that range. The sample where graphene was grown at 700 °C is offers the strongest absorption which corresponded to the best catalytic performance.

## Conclusions

The fabrication of graphene/TiO_2_/Ti hybrid film was demonstrated on a pure Ti foils through CVD method. Issues related to H2 absorption by the Ti matrix were partly alleviated by controlling the temperature ramping rate during graphene growth. By manipulating the growth conditions, the content of graphene and TiO_2_ can be controlled. The introduction of graphene to TiO_2_ and the formation of a high yield of vacancies in the form of Ti^3+^ ions within the matrix has strongly reinforced the photocatalytic properties of the hybrid material. Worth to mention, the use of film based photocatalyst is advantageous to the nanoparticle based system, as films can be easily applied and removed from the dye solution, minimising the concern of any surplus nanomaterials left in solution after photocatalytic treatment. The materials fabricated in this paper has provided practical application of graphene grown on pure Ti metals, which potentially expand the CVD graphene to a new metal member and explore the related applications.

## Electronic supplementary material

Supplementary Information 
