# Supplementary material for: Single step synthesis of Schottky-like hybrid graphene - titania interfaces for efficient photocatalysis
Source: Sci Rep. 2018 May 25;8:8154. doi: 10.1038/s41598-018-26447-9 (PMC5970265; doi:10.1038/s41598-018-26447-9)
Supplement: Supplementary file 1 — Supplementary Information [file 41598_2018_26447_MOESM1_ESM.pdf]

Supporting Information

Single step synthesis of Schottky-like hybrid graphene - titania interfaces for efficient photocatalysis

Zhifeng Yi[1,$*], Andrea Merenda[1], Lingxue Kong[1], Aleksandra Radenovic[2], Mainak Majumder[3], Ludovic F. Dumée[1,$]

[1] Deakin University, Geelong, Institute for Frontier Materials, Waurn Ponds 3216, Victoria, Australia

[2] Ecole Polytechnique Federale de Lausanne (EPFL), Institute of Biotechnology, CH-1015 Lausanne, Switzerland

[3] Department of Mechanical and Aerospace Engineering, Nanoscale Science and Engineering Laboratory (NSEL), Monash University, Clayton 3800, Victoria, Australia

Corresponding author:
Dr. Ludovic DUMEE (Ludovic.dumee@deakin.edu.au); +61410131312

[$] Both authors equally contributed to this manuscript.

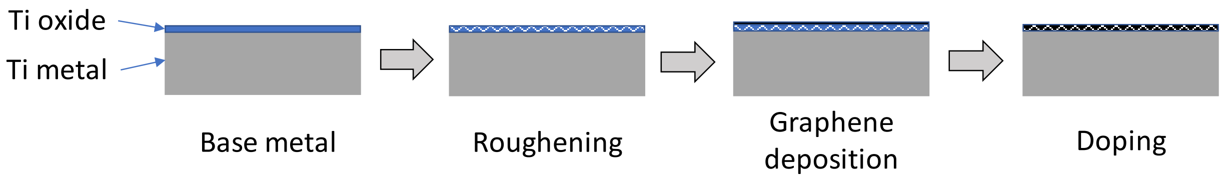

Figure S1 Schematic of the deposition and doping process

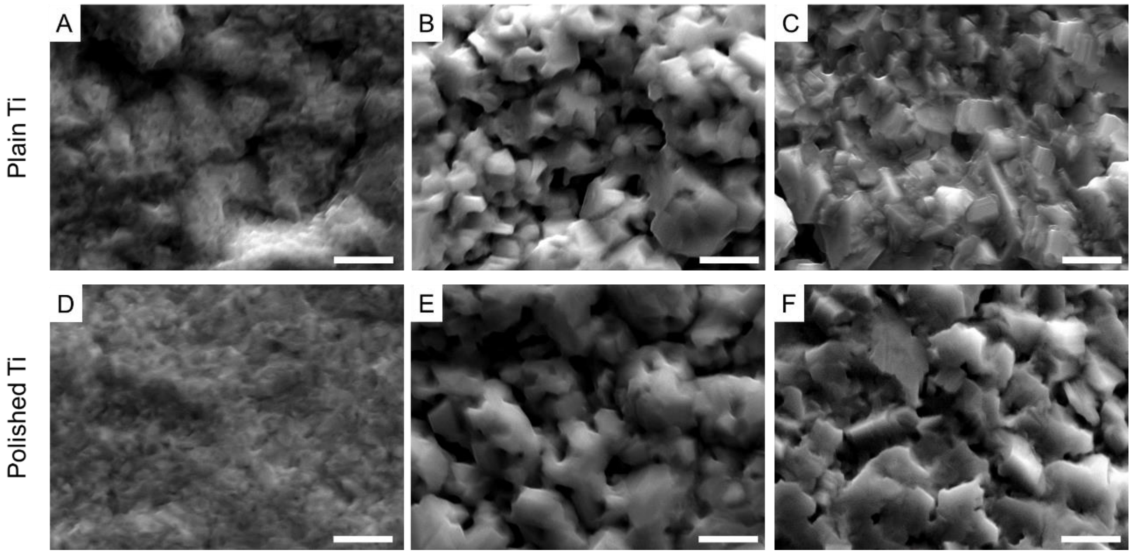

Figure S2 SEM images of plain and polished Ti foils heat treated at different temperatures. Plain Ti foils: (A) 700 ^{o}C, (B) 900 ^{o}C and (C) 1000 ^{o}C. Polished Ti foils: (D) 700 ^{o}C, (E) 900 ^{o}C and (F) 1000 ^{o}C.

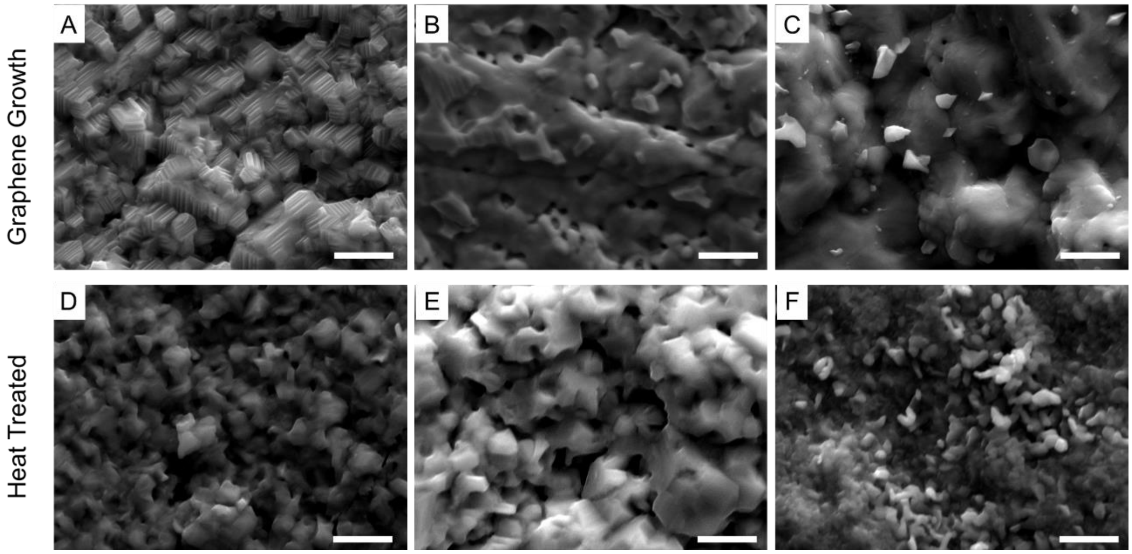

Figure S3 SEM images of CVD growth of graphene on plain Ti foils at 900 ^{o}C for 2 min (A), 5 min (B) and 10 min (C) and heat-treated samples at 900 ^{o}C for 2 min (D), 5 min (E) and 10 min (F).

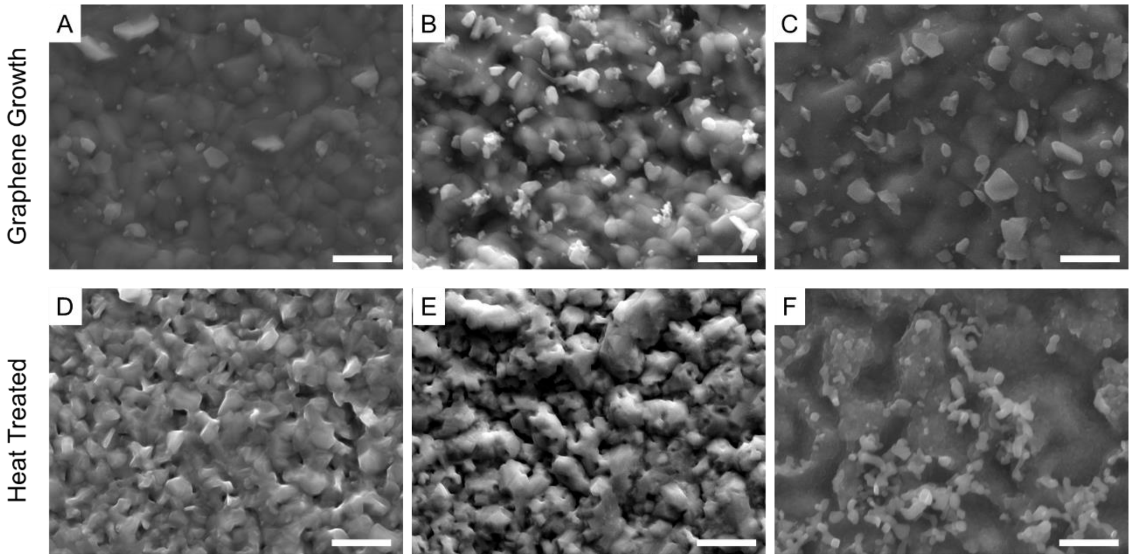

Figure S4 SEM images of CVD growth of graphene on polished Ti foils at 900 ^{o}C for 2 min (A), 5 min (B) and 10 min (C) and heat-treated samples at 900 ^{o}C for 2 min (D), 5 min (E) and 10 min (F).

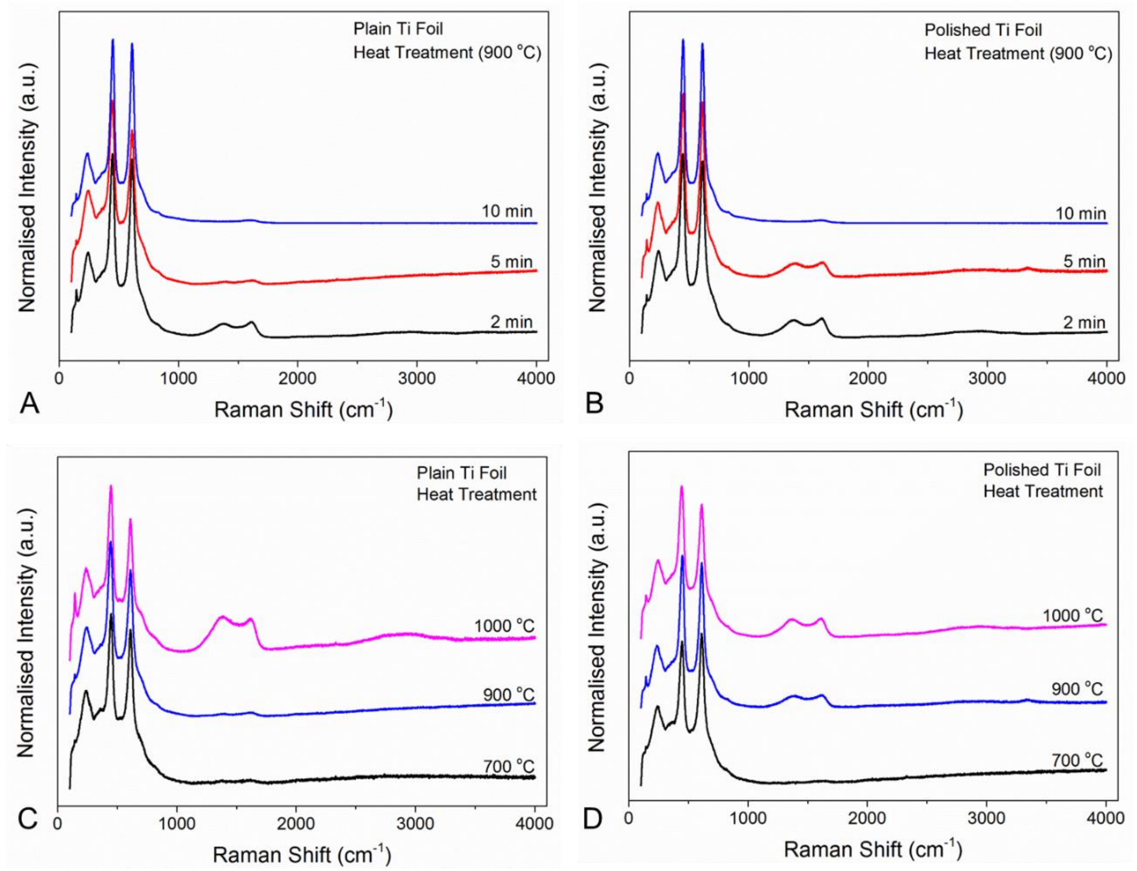

Figure S5 Raman spectra of Ti foils treated with different experimental conditions. Plain (A) and polished (B) Ti foils heat treated at 900 ^{o}C for 2, 5 and 10 min. Plain (C) and polished (D) Ti foils heat treated at different temperatures, 700-1000 ^{o}C, for 5 min.

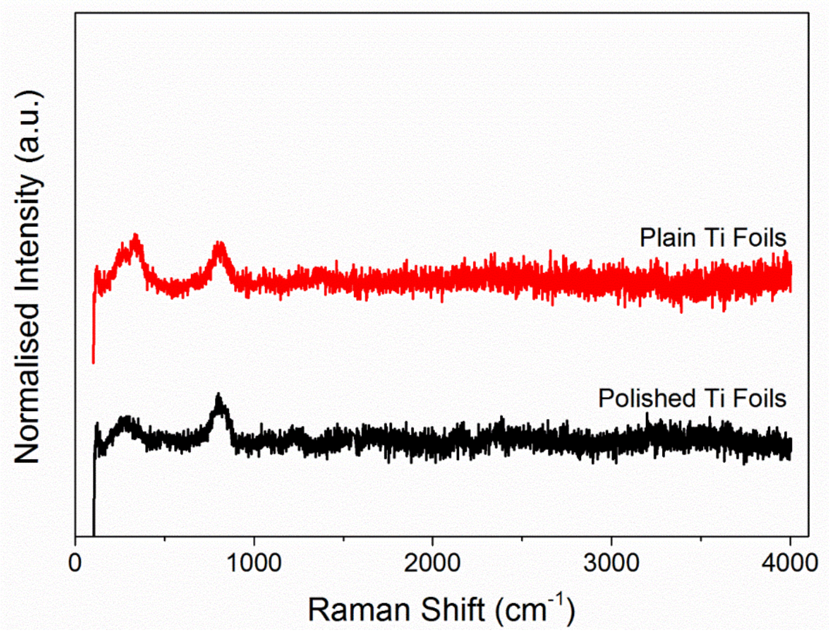

Figure S6 Raman spectra of plain and polished titanium foils.

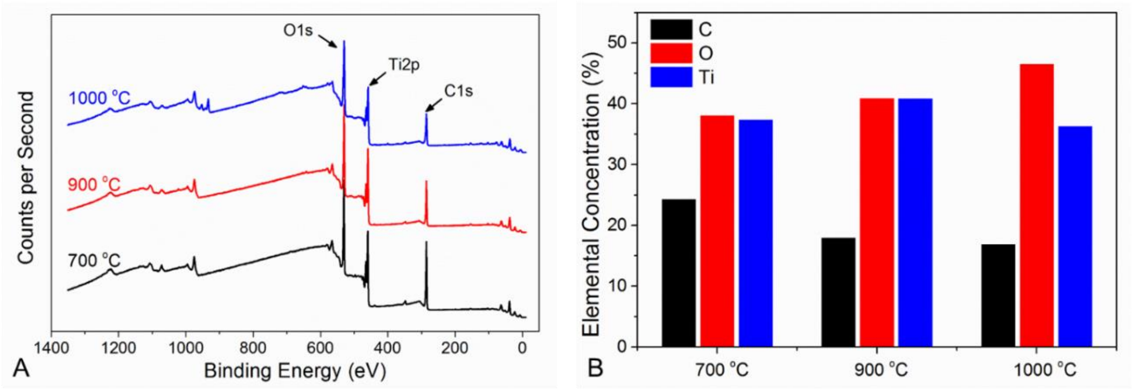

Figure S7 XPS survey scan (A) and the calculated elemental composition (B) of Ti foils with graphene growth at different temperatures.

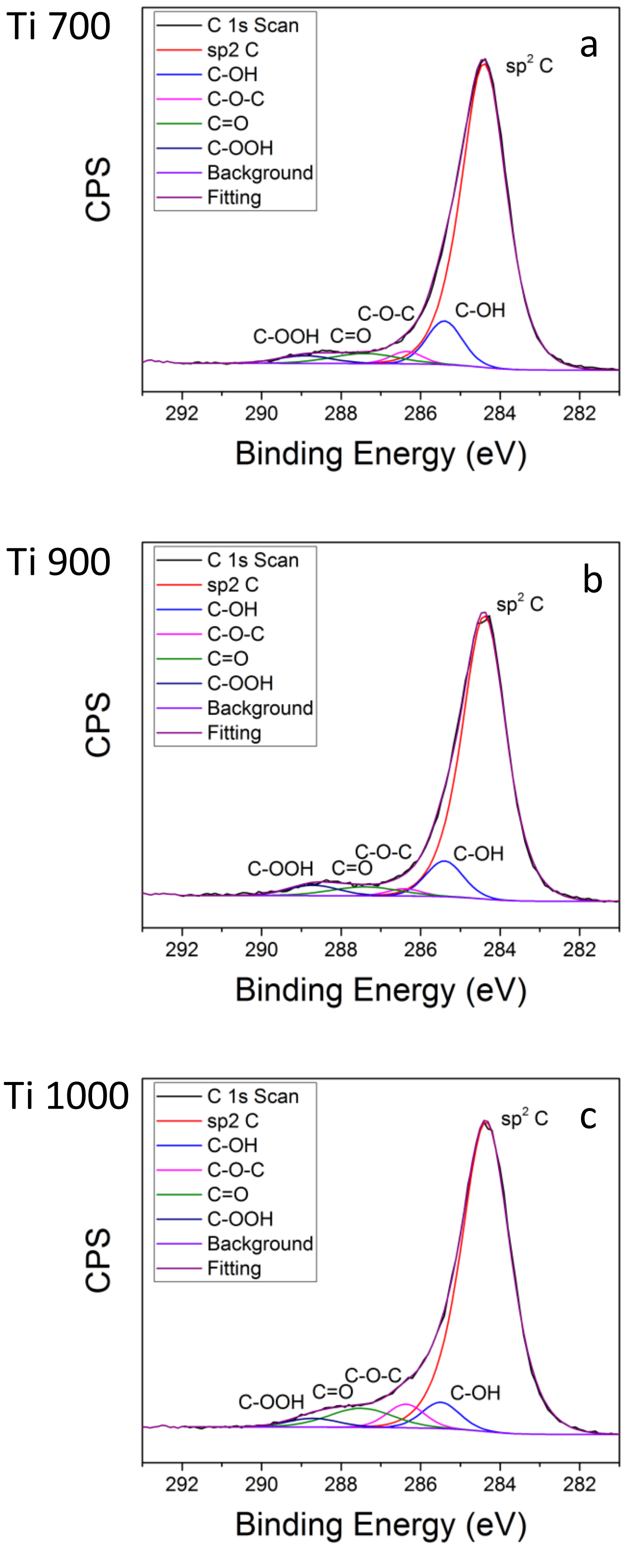

Figure S8 XPS C1S

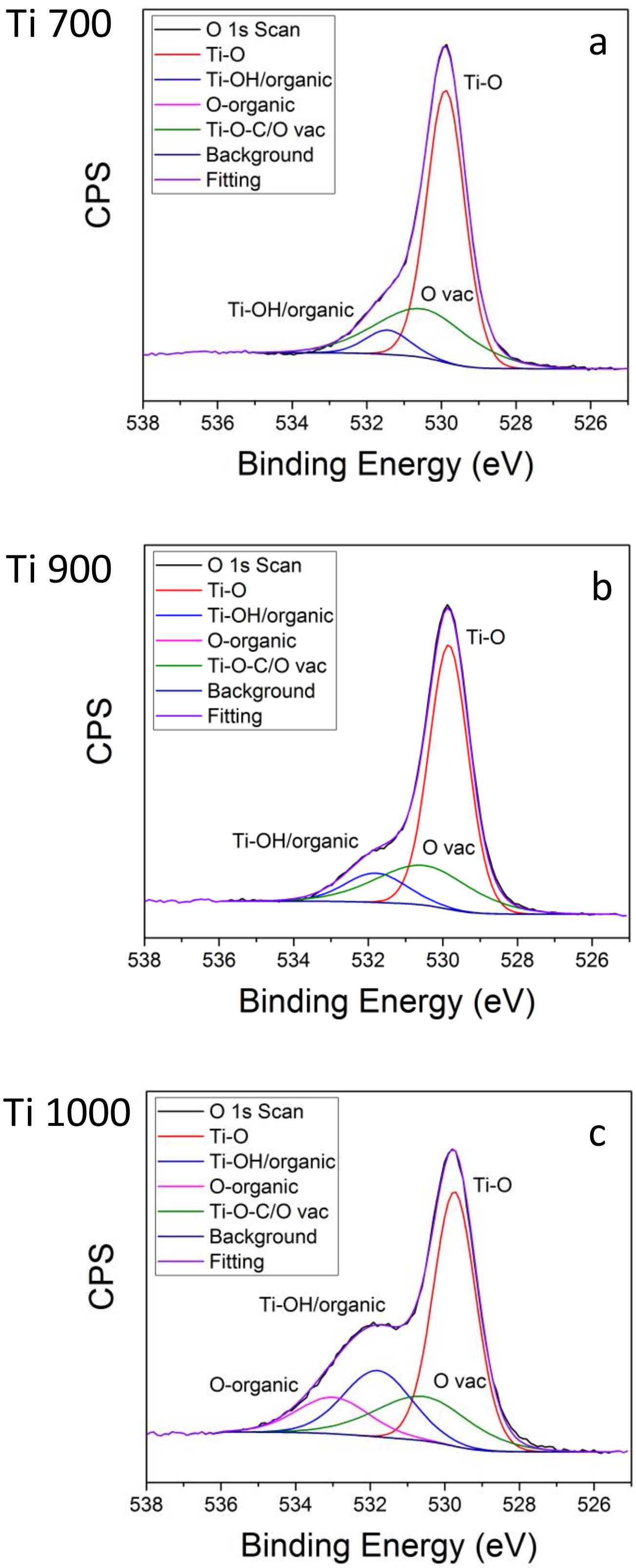

Figure S9 XP of the O1S and fittings for the series of samples

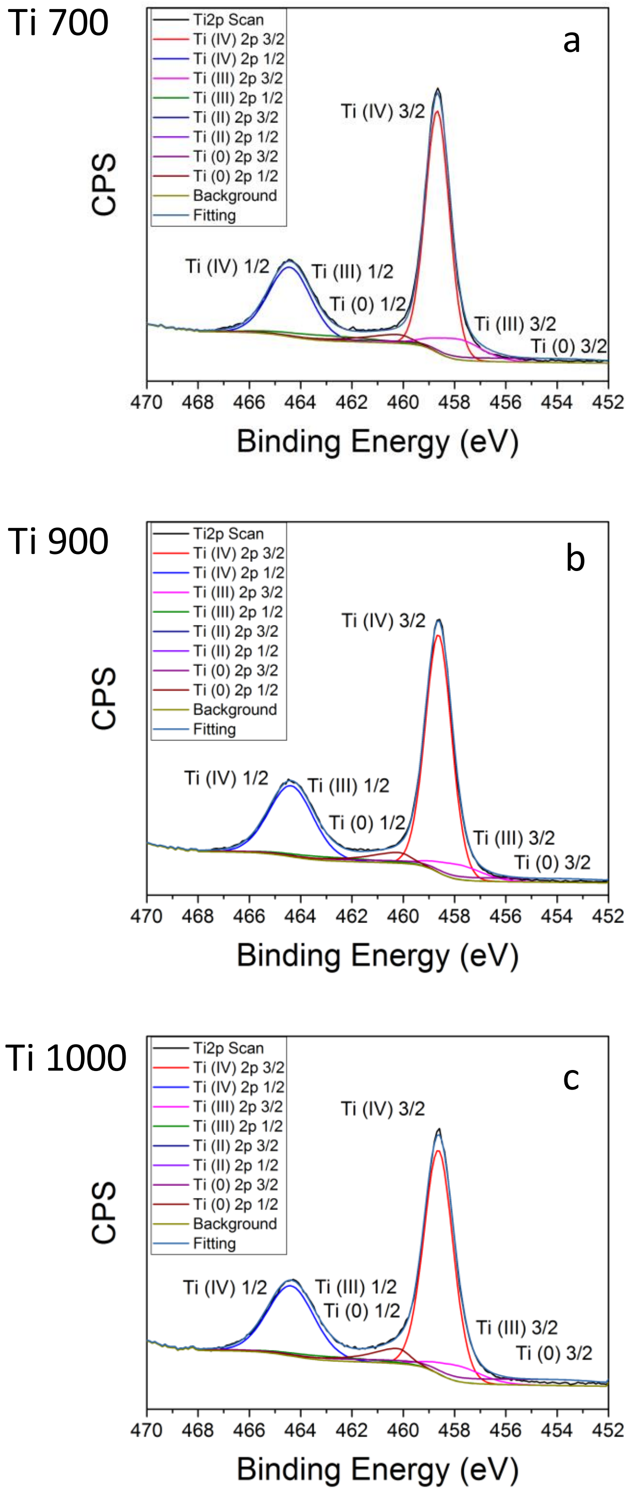

Figure S10 XPS of the Ti2p and fittings for the series of samples

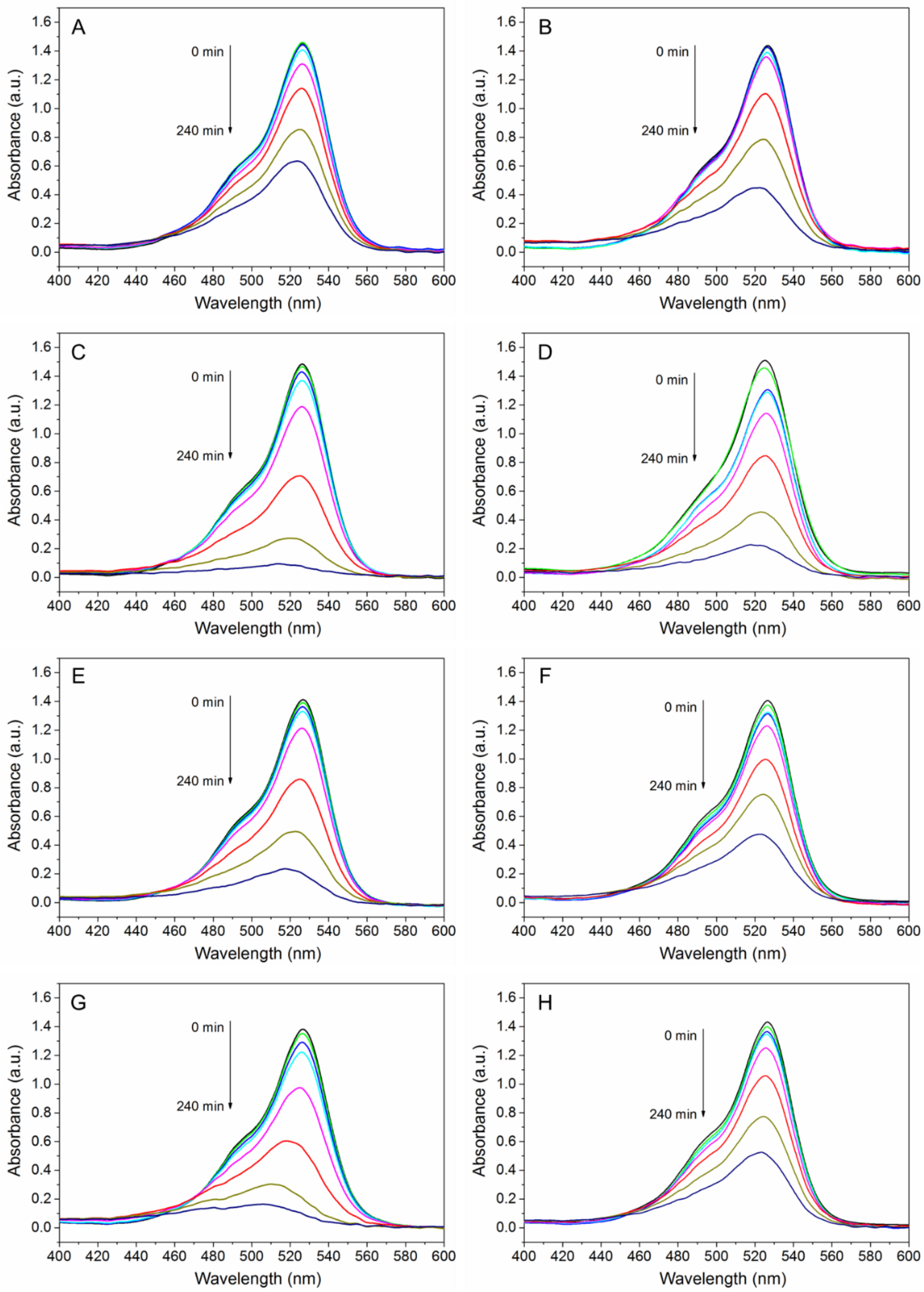

Figure S11 Photocatalytic performance of graphene Ti foils. (A) blank R6G solution exposed to UV light; (B) blank Ti foils. Ti foils with graphene fabricated at 700 ^{o}C (C), 900 ^{o}C (E) and 1000 ^{o}C (G). Ti foils treated at 700 ^{o}C (D), 900 ^{o}C (F) and 1000 ^{o}C (H).

Table S1. Evaluation of C 1s High Resolution spectra and relative position and FWHM of C components.

**Component**	**700**		**900**		**1000**	
	Position (eV)	FWHM (eV)	Position (eV)	FWHM (eV)	Position (eV)	FWHM (eV)
**sp2 C**	284.4	1.327	284.39	1.294	284.35	1.436
**C-OH**	285.4	1.051	285.4	1.1	285.5	1.111
**C-O-C**	286.35	0.942	286.4	1	286.37	1.1
**C=O**	287.4	1.976	287.4	2	287.5	1.904
**C-OOH**	288.9	1.576	288.7	1.5	288.7	1.497

Table S2 Average values for C components evaluated by C 1s spectra.

	**Average**			
	Position (eV)	Standard Deviation	FWHM (eV)	Standard Deviation
**sp2 C**	284.4	0.02	1.35	0.06
**C-OH**	285.4	0.05	1.09	0.03
**C-O-C**	286.4	0.02	1.01	0.07
**C=O**	287.4	0.05	1.96	0.04
**C-OOH**	288.8	0.09	1.52	0.04

Table S3 Evaluation of O 1s High Resolution spectra and relative position and FWHM of O components.

**Component**	**700**		**900**		**1000**	
	Position (eV)	FWHM (eV)	Position (eV)	FWHM (eV)	Position (eV)	FWHM (eV)
**O (metallic)**	529.88	1.196	529.84	1.237	529.74	1.347
**O (OH, organic)**	531.44	1.392	531.8	1.928	531.8	2.14
**O (organic)**	533	5	533	5	533	5
**O vac**	530.5	2.984	530.5	3	530.57	3

Table S4 Average values for O components evaluated by O 1s spectra.

**Component**	**Average**			
	Position (eV)	Standard Deviation	FWHM (eV)	Standard Deviation
**O (metallic)**	265.5	0.06	1.26	0.00
**O (OH, organic)**	266.8	0.17	1.82	0.07
**O (organic)**	269.0	0.00	5.00	0.00
**O vac**	266.8	0.03	2.99	0.01

Table S5. Evaluation of Ti 2p High Resolution spectra and relative position and FWHM of Ti components.

**Component**	**700**		**900**		**1000**	
	Position (eV)	FWHM (eV)	Position (eV)	FWHM (eV)	Position (eV)	FWHM (eV)
**Ti (IV) 2p 3/2**	458.67	1.106	458.63	1.207	458.63	1.336
**Ti (iV) 2p 1/2**	464.42	1.926	464.38	2.025	464.38	2.132
**Ti (III) 2p 3/2**	457.9	2.2	457.9	2.2	457.9	2.2
**Ti (III) 2p 1/2**	463.1	4.435	463.1	4.435	463.1	4.4
**Ti (II) 2p 3/2**	455	2.21	455	2.21	455	2.21
**Ti (II) 2p 1/2**	460.6	0.739	460.6	0.739	460.6	0.739
**Ti (0) 2p 3/2**	454.1	5.764	454.07	5.734	454.1	5.556
**Ti (0) 2p 1/2**	460.2	1.327	460.17	1.217	460.2	1.1

Table S6. Average values for Ti components evaluated by Ti 2p spectra.

**Component**	**Average**			
	Position (eV)	Standard Deviation	FWHM (eV)	Standard Deviation
**Ti (IV) 2p 3/2**	229.93	0.02	1.22	0.04
**Ti (iV) 2p 1/2**	233.21	0.02	2.03	0.03
**Ti (III) 2p 3/2**	230.1	0.00	2.20	0.00
**Ti (III) 2p 1/2**	233.8	0.00	4.42	0.01
**Ti (II) 2p 3/2**	228.6	0.00	2.21	0.00
**Ti (II) 2p 1/2**	230.7	0.00	0.74	0.00
**Ti (0) 2p 3/2**	229.9	0.01	5.68	0.04
**Ti (0) 2p 1/2**	230.7	0.01	1.21	0.04
